# Increased drought tolerance in plants engineered for low lignin and low xylan content

**DOI:** 10.1186/s13068-018-1196-7

**Published:** 2018-07-18

**Authors:** Jingwei Yan, Aude Aznar, Camille Chalvin, Devon S. Birdseye, Edward E. K. Baidoo, Aymerick Eudes, Patrick M. Shih, Dominique Loqué, Aying Zhang, Henrik V. Scheller

**Affiliations:** 1 Joint BioEnergy Institute and Environmental Genomics and Systems Biology Division, Lawrence Berkeley National Laboratory, Berkeley, CA 94720 USA; 2 College of Life Sciences, Nanjing Agricultural University, Nanjing, 210095 Jiangsu China; 3 Ecole Normale Supérieure de Cachan, 94230 Cachan, France; 4 Joint BioEnergy Institute and Biological Systems and Engineering Division, Lawrence Berkeley National Laboratory, Berkeley, CA 94720 USA; 5 Department of Plant and Microbial Biology, University of California, Berkeley, Berkeley, CA 94720 USA

**Keywords:** Drought tolerance, Abscisic acid, Cell walls, Lignin, Xylan, Biofuel, Synthetic biology, *Arabidopsis thaliana*

## Abstract

**Background:**

We previously developed several strategies to engineer plants to produce cost-efficient biofuels from plant biomass. Engineered Arabidopsis plants with low xylan and lignin content showed normal growth and improved saccharification efficiency under standard growth conditions. However, it remains to be determined whether these engineered plants perform well under drought stress, which is the primary source of abiotic stress in the field.

**Results:**

Upon exposing engineered Arabidopsis plants to severe drought, we observed better survival rates in those with a low degree of xylan acetylation, low lignin, and low xylan content compared to those in wild-type plants. Increased pectic galactan content had no effect on drought tolerance. The drought-tolerant plants exhibited low water loss from leaves, and drought-responsive genes (*RD29A*, *RD29B*, *DREB2A*) were generally up-regulated under drought stress, which did not occur in the well-watered state. When compared with the wild type, plants with low lignin due to expression of QsuB, a 3-dehydroshikimate dehydratase, showed a stronger response to abscisic acid (ABA) in assays for seed germination and stomatal closure. The low-lignin plants also accumulated more ABA in response to drought than the wild-type plants. On the contrary, the drought tolerance in the engineered plants with low xylan content and low xylan acetylation was not associated with differences in ABA content or response compared to the wild type. Surprisingly, we found a significant increase in galactose levels and sugar released from the low xylan-engineered plants under drought stress.

**Conclusions:**

This study shows that plants engineered to accumulate less lignin or xylan are more tolerant to drought and activate drought responses faster than control plants. This is an important finding because it demonstrates that modification of secondary cell walls does not necessarily render the plants less robust in the environment, and it shows that substantial changes in biomass composition can be achieved without compromising plant resilience.

**Electronic supplementary material:**

The online version of this article (10.1186/s13068-018-1196-7) contains supplementary material, which is available to authorized users.

## Background

The plant cell wall is a complex composite of cellulose, various hemicelluloses, pectic polysaccharides, and the aromatic polymer lignin, all of which play critical roles in plant growth, defense, and morphology [1]. Besides that, cell walls also constitute the most abundant biomaterial on earth and hold the potential to provide a renewable source for biofuel production [2–4]. However, biofuels derived from lignocellulosic biomass are not currently cost competitive with petroleum. This is largely due to the facts that (1) the presence of lignin in the cell wall makes it recalcitrant to enzymatic hydrolysis, (2) a low hexose/pentose ratio (most hexoses can be more easily fermented by yeast into fuels than pentoses), and (3) the presence in biomass of inhibitors of fermentation such as acetate [5, 6]. Therefore, our current research is aimed at enhancing polysaccharide accumulation in raw biomass, improving the biomass digestibility, and increasing the hexose/pentose ratio [7–12].

Xylans, the main component of hemicellulose in secondary cell walls, are composed almost entirely of pentose sugars and are esterified with acetate, which hinders the enzymatic saccharification of wall polymers [13, 14]. Thus, plants with reduced amounts of xylan and xylan acetylation in the secondary cell wall are considered better feedstocks for biofuel production [8, 11]. It has been demonstrated that using a vessel-specific complementation of Arabidopsis mutants deficient in xylan biosynthesis maintains the lower xylan content while increasing the saccharification yield and hexose/pentose ratio [8]. TBL29 is a xylan acetyltransferase [15, 16]. While Arabidopsis *tbl29* mutants are severely dwarfed, engineered plants expressing the *AtGUX1* xylan glucuronosyltransferase under a native *TBL29* promoter in the *tbl29* mutant background exhibited a normal growth phenotype compared to the wild type and showed a significant decrease in the degree of acetylation of xylan [17]. Lignin in plant biomass is the main contributor to cell wall recalcitrance, thus low lignin can substantially improve the saccharification efficiency of plant cell walls [18, 19]. However, most efforts to decrease lignin content resulted in severe biomass yield reduction [20, 21]. In a recent study we reported that expression of a 3-dehydroshikimate dehydratase (QsuB from *Corynebacterium glutamicum*), driven by a *C4H* promoter, results in dramatically reduced lignin content and improved saccharification efficiency without impacting plant growth [22]. Finally, research has been aimed at improving hexose content by increasing β-1,4-galactan in biomass. β-1,4-galactan is synthesized by GALS galactan synthase enzymes but overexpression of *GALS1* in Arabidopsis did not increase stem galactose content [23]. In contrast, simultaneous overexpression of *AtUGE2* (UDP-glucose epimerase) and *GALS1* increases the stem cell wall galactose content, providing a promising method of engineering advanced feedstocks for biofuel [10]. Since the URGT1 UDP-galactose transporter appeared to be limiting for β-1,4-galactan accumulation [10, 24], we recently combined overexpression of *URGT1* with *UGE2* and *GALS1* [12]. We also stacked the high galactan trait with decreased lignin and decreased xylan.

These engineered Arabidopsis plants show excellent growth in the laboratory [8–10, 12, 17, 22], but it is important to assess how they will perform in the natural environment where they are frequently exposed to unfavorable environmental stress. It has been estimated that the yield of field-grown crops in the United States is only 22% of the genetic potential yield [25]. Among such varied stresses, water deficiency affects the largest fraction (25.3%) of the US land surface [25]. Thus, it is important to understand the physiological processes that underlie stress injury and the adaptation of cell wall modified plants to drought stress. Surprisingly, this question has received very little attention.

In the present study, we tested under drought stress the phenotype of several engineered lines with altered cell wall composition. We found that the plants engineered for low lignin content confer the adaptation to drought tolerance by more rapidly elevating ABA levels and positively regulating the expression of drought-responsive genes. The plants engineered for low xylan content and a low acetyl substitution degree of xylan have improved drought tolerance in an ABA-independent manner. Surprisingly, we found that the plants engineered for low xylan content have significantly increased saccharification efficiency under drought stress. This allows us to generate drought-resistant engineered plants, which also show a high sugar release from the biomass under environmental stress conditions.

## Results

### Engineered plants with low degree of acetylation of xylan, low lignin, and low xylan have improved tolerance to drought

**Fig. 1:**
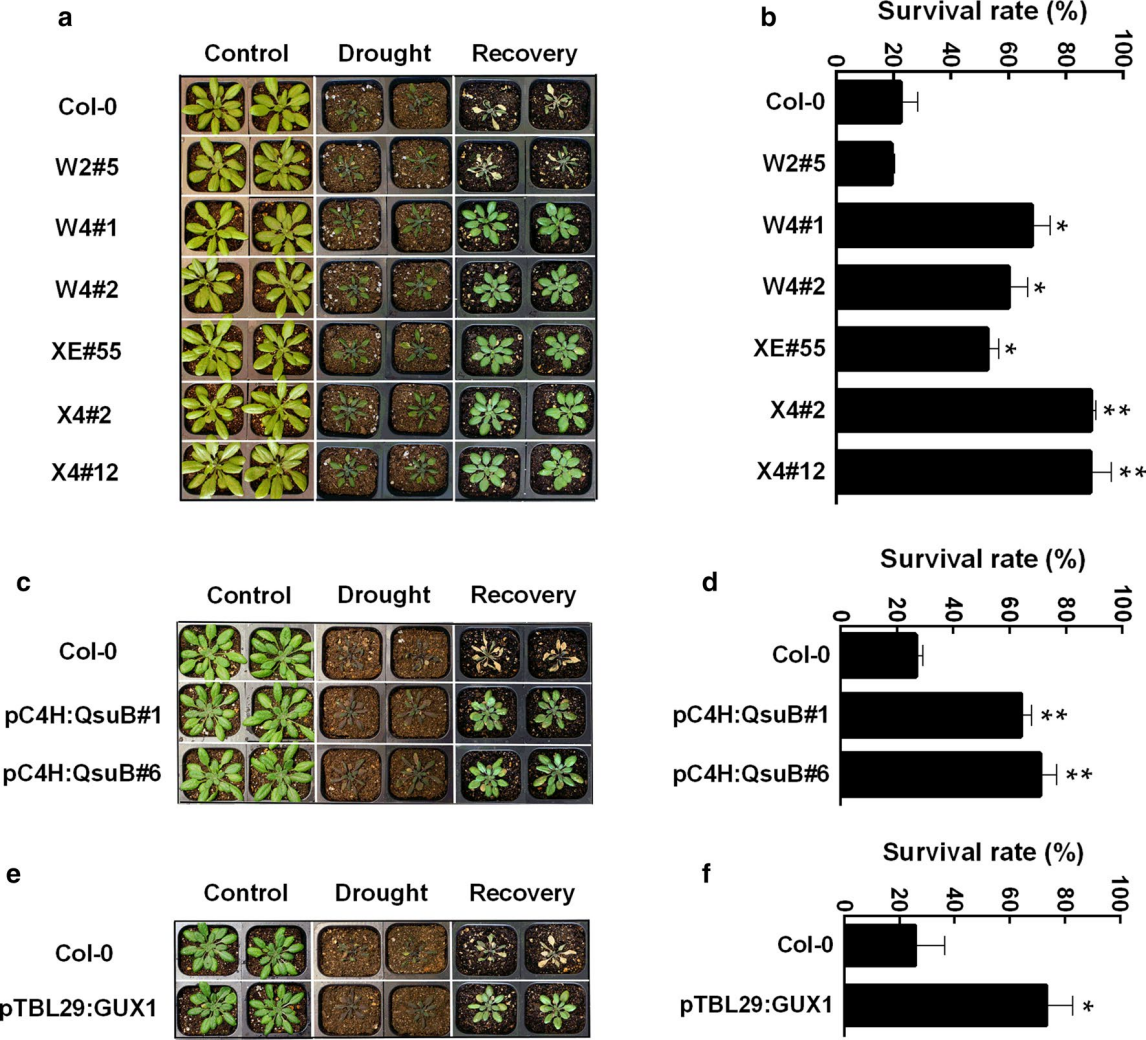
The phenotype of Arabidopsis engineered plants W2#4, W4#1, W4#2, XE#55, X4#2, X4#12, pC4H:QsuB#1, pC4H:QsuB#6 and pTBL29:GUX1 compared with wild type under normal and drought stress condition on soil. **a** Phenotype of Arabidopsis engineered plants W2#4, W4#1, W4#2, XE#55, X4#2 and X4#12 compared with wild type (Col-0) under normal and drought stress condition. **b** Corresponding survival rates. **c** Phenotype of Arabidopsis engineered pC4H:QsuB#1 and pC4H:QsuB#6 compared with wild type (Col-0) under normal and drought stress condition. **d** Corresponding survival rates. **e** Phenotype of Arabidopsis engineered pTBL29:GUX1 compared with wild type (Col-0) under normal and drought stress condition. **f** Corresponding survival rates. Values in (**b**, **d** and **f**) show average ± SD (*n* = 3). The experiments in (**a**, **c** and **e**) were repeated at least three times and at least 35 plants for each genotype were assessed for the survival rate in each experiment. Asterisks indicate significant differences from the wild type (*t* test, *P < 0.05; **P < 0.01)

Several engineered Arabidopsis lines that showed normal growth under well-watered conditions (Table 1) were tested to investigate their response to drought stress conditions. The plants have altered content of galactan, xylan, lignin and/or acetylated xylan as shown in the original studies [8, 12, 17, 22] and are summarized in Table 1. Three-week-old plants were subjected to progressive levels of drought by withholding water for 14 days. As shown in Fig. 1, all of the engineered lines showed comparable growth vigor under the well-watered condition, in agreement with what was previously described [8, 12, 17, 22]. Interestingly, the engineered lines XE#55, pC4H:QsuB#1, pC4H:QsuB#6, W4#1, W4#2, X4#2, X4#12, and pTBL29:GUX1 showed fewer wilting symptoms after 14 days of withholding water compared to wild-type (Col-0) and W2#5 plants (Fig. 1). These engineered plants, except for the high galactan W2#5 plants, were significantly better able to survive than the wild type after recovering with water for 2 days (Fig. 1). More than 50% of XE#55, pC4H:QsuB#1, pC4H:QsuB#6, W4#1, W4#2, and pTBL29:GUX1 plants were recovered compared to only 20% of Col-0. The survival rate of X4#2 and X4#12 lines, which combine low lignin and low xylan, reached 80% (Fig. 1). This showed that the engineered plants with low xylan, low lignin, and a low acetyl substitution degree of xylan were more drought-tolerant than wild-type and W2#5 plants. The increased survival rate of plants under water deficit can be associated with the capacity to maintain lower water loss, and indeed we observed a reduced rate of water loss from detached leaves in all the plants with increased survival (Additional file 1).

**Table 1:** Features and nomenclature of engineered Arabidopsis plants used in this study

Name	Background	Genotype	Phenotype	References
pTBL29:GUX1	*tbl29*	*pTBL29:GUX1*	Low xylan acetylation	[17]
XE#55^a^	*irx7*	*pVND7:IRX7*	Low xylan in fibers	[8]
pC4H:QsuB#1	Col-0	*pC4H:QsuB*	Low lignin, high H/G ratio	[22]
pC4H:QsuB#6	Col-0	*pC4H:QsuB*	Low lignin, high H/G ratio	[22]
W2#5	Col-0	*pC4H:GALS1* *pIRX5:UGE2* *pIRX8:URGT1*	High β-1,4-galactan. High C6/C5 ratio	[12]
W4#1	Col-0	*pC4H:GALS1* *pIRX5:UGE2* *pIRX8:URGT1* *pCESA7:QsuB*	High β-1,4-galactan. High C6/C5 ratio. Low lignin	[12]
W4#2	Col-0	*pC4H:GALS1* *pIRX5:UGE2* *pIRX8:URGT1* *pCESA7:QsuB*	High β-1,4-galactan. High C6/C5 ratio. Low lignin	[12]
X4#2	XE#55^a^	*pC4H:GALS1* *pIRX5:UGE2* *pIRX8:URGT1* *pCESA7:QsuB*	High β-1,4-galactan. High C6/C5 ratio. Low lignin. Low xylan	[12]
X4#12	XE#55^a^	*pC4H:GALS1* *pIRX5:UGE2* *pIRX8:URGT1* *pCESA7:QsuB*	High β-1,4-galactan. High C6/C5 ratio. Low lignin. Low xylan	[12]

The nomenclature and the line numbers are the same as used in the referenced studies

^a^ The name used for this line is the same as in the first study [8]. The XE#55 line was used as a control with the designation ‘X0’ in the recent paper by Aznar et al. [12]

Most of the results reported here were obtained with plants in the rosette stage. However, similar levels of drought tolerance were observed with older plants that had already bolted and flowered during the drought treatment (Additional file 2).

The engineered lines were also tested for their response to osmotic stress by transferring 5-day-old seedlings to 1/2 MS medium containing 0, 300, and 400 mM mannitol, and allowing them to grow for 10 days. As shown in Additional file 3, no noticeable difference was observed in the growth of these plants under osmotic stress.

### Engineered plants with low lignin have increased ABA sensitivity

**Fig. 2:**
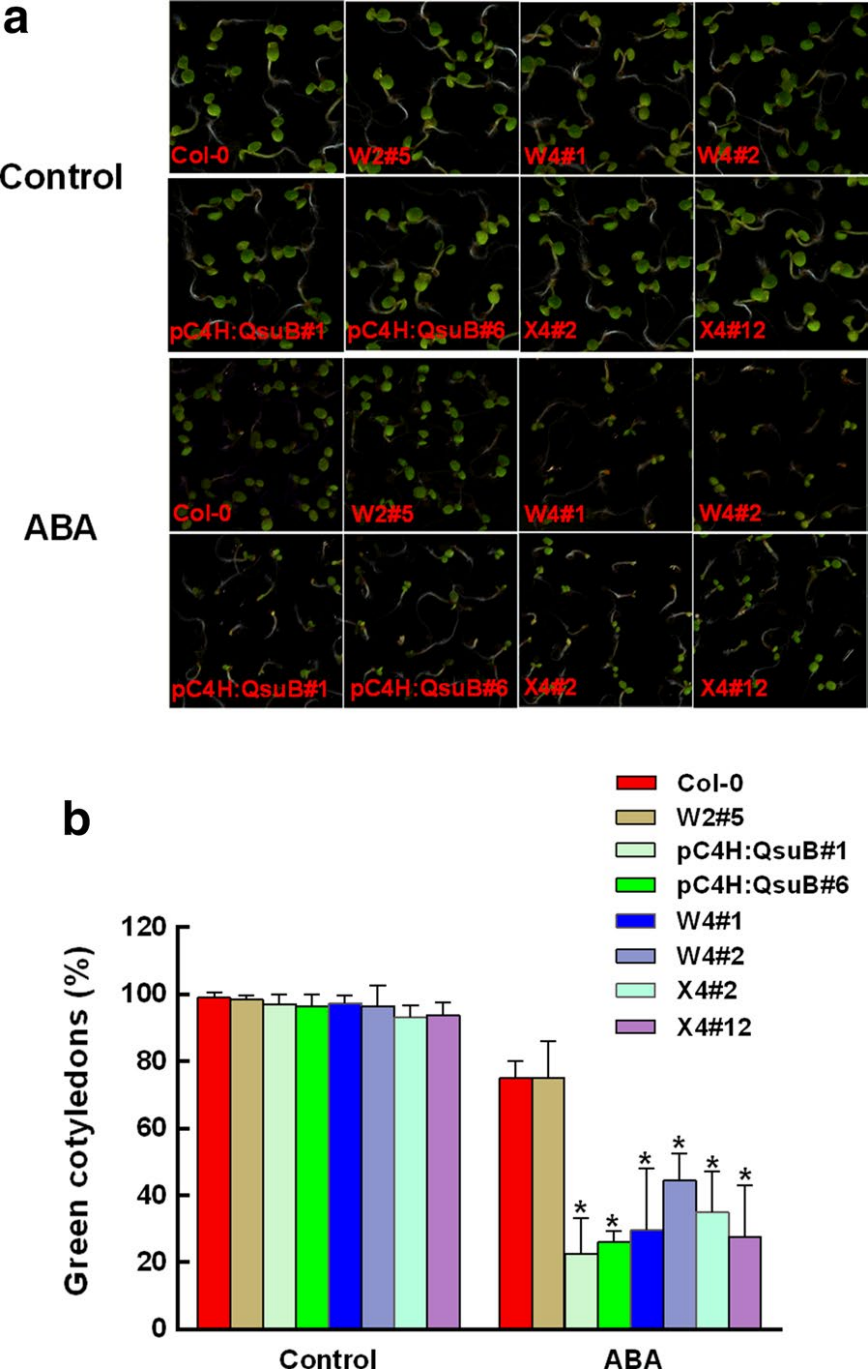
Germination of the engineered plants in response to exogenous ABA. **a** The wild-type and engineered plants were sowed on 1/2 MS medium containing 0 and 0.5 µM ABA. Photographs were taken after 6 days. **b** The percentage of seedlings with green cotyledons was measured after 6 days. Each experiment included at least 100 seeds per genotype, and three independent experiments were conducted. Asterisks indicate significant differences from the wild type (*t* test, *P < 0.05)

ABA is an essential mediator in triggering plant responses to most of the common abiotic stresses, including drought, salinity, high temperature, and oxidative stress [26–28]. To further investigate whether the drought tolerance of the engineered Arabidopsis plants was ABA dependent, we first studied the cotyledon greening under exogenous ABA treatment. In the absence of exogenous ABA, the engineered lines and wild type had similar cotyledon greening rates. In the presence of 0.5 µM ABA, pC4H:QsuB#1, pC4H:QsuB#6, W4#1, W4#2, X4#2, and X4#12 lines, which all have a low lignin content, showed obvious decreases in the cotyledon greening percentages compared with the wild type, whereas the W2#5 plants, which have high galactan but no engineering of lignin or xylan, did not differ from the wild type (Fig. 2). Furthermore, the cotyledon greening rates of pTBL29:GUX1 and XE#55 were also the same as in the wild type (Additional file 4). These results implied that the drought tolerance of engineered plants with low lignin is somehow related to ABA, whereas the tolerance of engineered plants with low xylan or low xylan acetylation is ABA independent.

### Engineered plants with low lignin have increased ABA level under drought stress

**Fig. 3:**
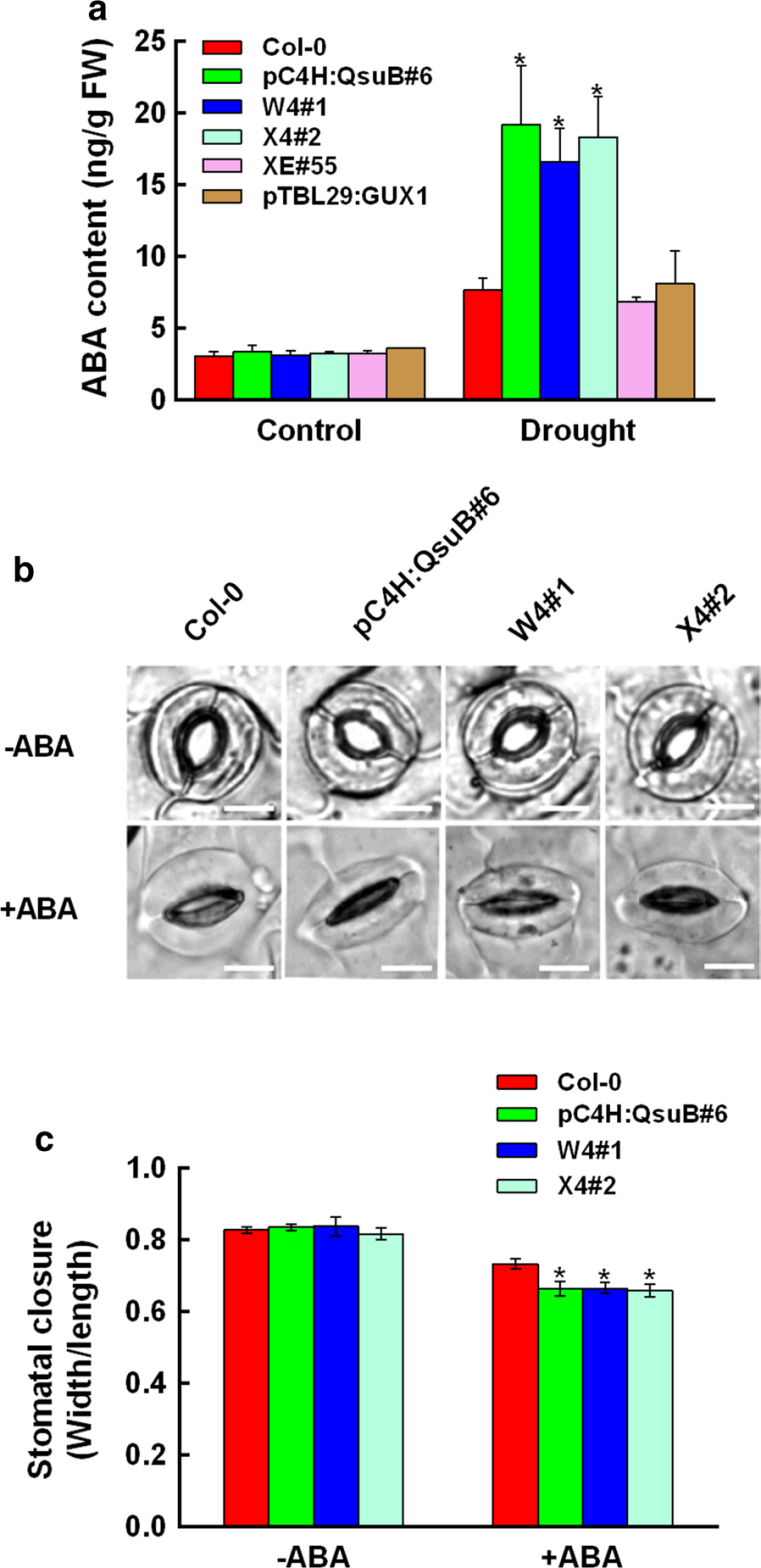
Quantification of endogenous ABA content and analysis of ABA-induced stomatal closure. **a** Determination of endogenous ABA level in Col-0 and engineered plant leaves after drought treatments. **b** Photo of the stomatal aperture in Col-0 and engineered plants for low lignin content (W4#1, pC4H:QsuB#6 and X4#12) under 10 µM ABA treatment. Bars = 10 µm. **c** The ratios of width to length of the stomatal aperture in response to 10 µM ABA treatment. Values in (**a** and **c**) show average ± SD (*n* = 3). At least 50 stomata were measured for each genotype per replication. Asterisks indicate significant differences from the wild type (*t* test, *P < 0.05)

We also considered the possibility that cell wall perturbations in water-conducting tissues may lead to increased ABA levels and activation of stress responses even in the well-watered state, as has been observed in several irregular xylem mutants. The engineered plants with low-lignin pC4H:QsuB, W4 and X4 showed increased drought tolerance (Fig. 1), but under well-watered conditions, there was no difference in ABA content between these engineered plants and the wild type. This result indicates that no drought response was activated prior to the drought treatment (Fig. 3a). However, when the plants were exposed to drought stress by withholding water for 9 days, the endogenous ABA contents of pC4H:QsuB#6, W4#1, and X4#12 were more than twice as high as in the wild type (Fig. 3a). In contrast, the ABA level of pTBL29:GUX1 and XE#55 under drought stress was similar to that of the wild type (Fig. 3a). This result further indicates that the increased drought tolerance of the engineered plants with low lignin is related to ABA, whereas the modified-xylan traits cause increased drought tolerance in an ABA-independent manner.

### Engineered plants with low lignin have enhanced ABA sensitivity in stomatal closure

When plants are exposed to drought stress, stomata close to minimize water loss [29]. ABA plays a vital role in this process. We further investigated whether the stomatal apertures were affected, given that endogenous ABA levels in response to drought were up-regulated in pC4H:QsuB, W4, and X4 compared to wild type (Fig. 3a). As shown in Fig. 3b and c, stomatal aperture under non-stressed conditions was similar in all plants, but in the presence of exogenous ABA, stomatal aperture was reduced in engineered pC4H:QsuB#6, W4#1, and X4#12. This indicated that the stomatal closures in engineered plants with low lignin are more responsive to ABA compared to wild type, which may be critical for adapting to drought stress.

### Engineered plants with low acetyl substitution of xylan, low lignin, and low xylan up-regulate the expression of drought-responsive genes

**Fig. 4:**
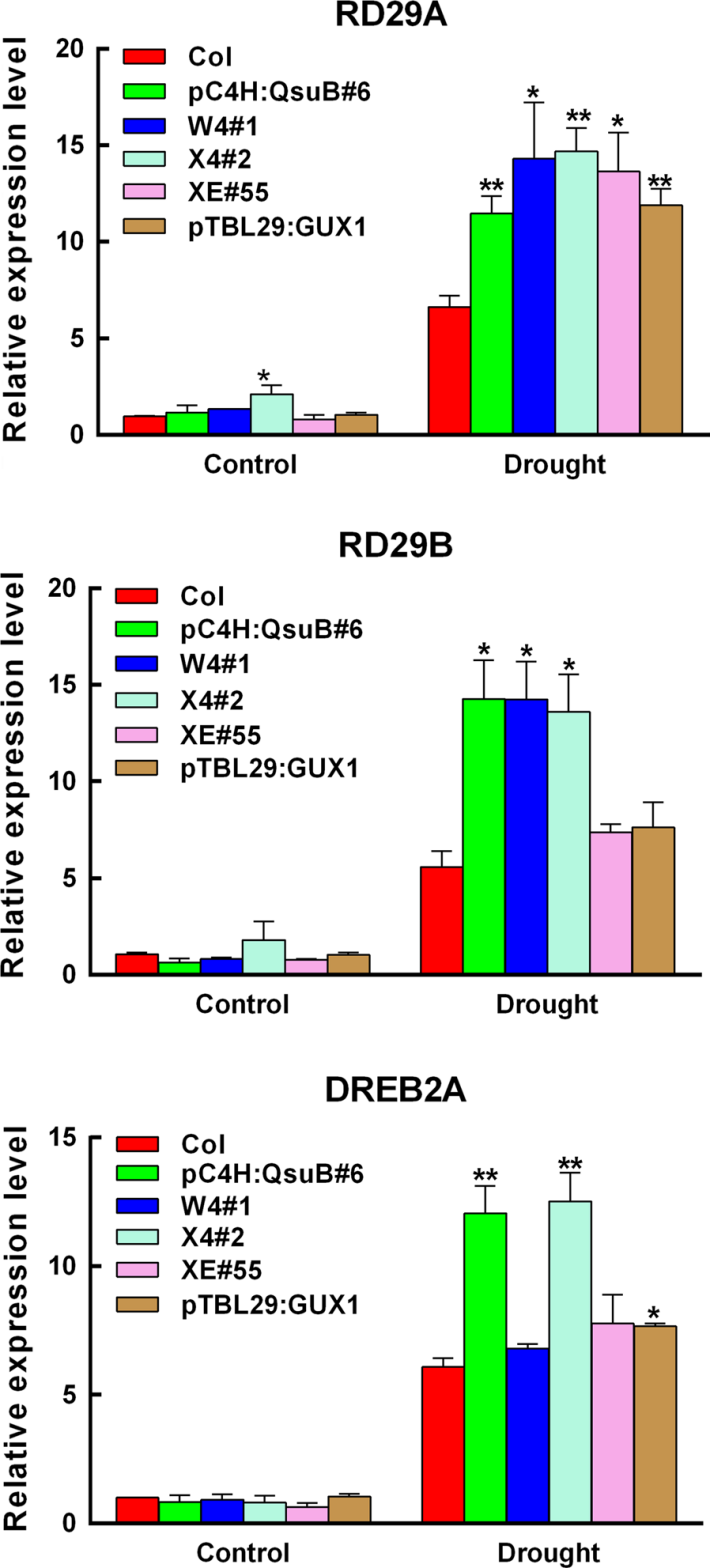
Expression of drought-responsive genes in the wild-type and engineered Arabidopsis plants in response to drought treatment. Relative expression levels of *RD29A*, *RD29B* and *DREB2A*. Total RNA was isolated from rosettes grown with water withholding for 9 days and subjected to quantitative reverse transcription-polymerase chain reaction (qRT-PCR) analysis. Values show average ± SD (*n* = 3). Asterisks indicate significant differences from the wild type (*t* test, *P < 0.05; **P < 0.01)

To gain further insight into the molecular mechanism underlying enhanced drought tolerance in the engineered plants, the expression of some drought-responsive genes was examined in the engineered and wild-type plants under drought stress. We chose the *RD29A*, *RD29B,* and *DREB2A* genes as stress-responsive markers [30, 31]. As shown in Fig. 4, the expression of *RD29A*, *RD29B*, and *DREB2A* under non-stressed conditions was similar in all plants except for the expression of *RD29A* in X4#2, which has a twofold increase compared to wild type. In response to drought, the expression of all three genes increased dramatically in all the plants, including the wild type. However, some of the engineered plants showed much higher induction than the wild type. Expression of *RD29A* was up-regulated compared to wild type in all the engineered plants, the *RD29B* expression was only increased in the low lignin plants (pC4H:QsuB#6, W4#1 and X4#2), and the *DREB2A* expression was only substantially up-regulated in the engineered pC4H:QsuB#6 and X4#2 lines (Fig. 4). These results indicate that plants engineered for low acetyl substitution of xylan, low lignin, and low xylan have increased induction of stress-responsive genes, which may account for their enhanced drought tolerance.

### Biomass from engineered plants with low xylan showed improved saccharification under drought stress

**Fig. 5:**
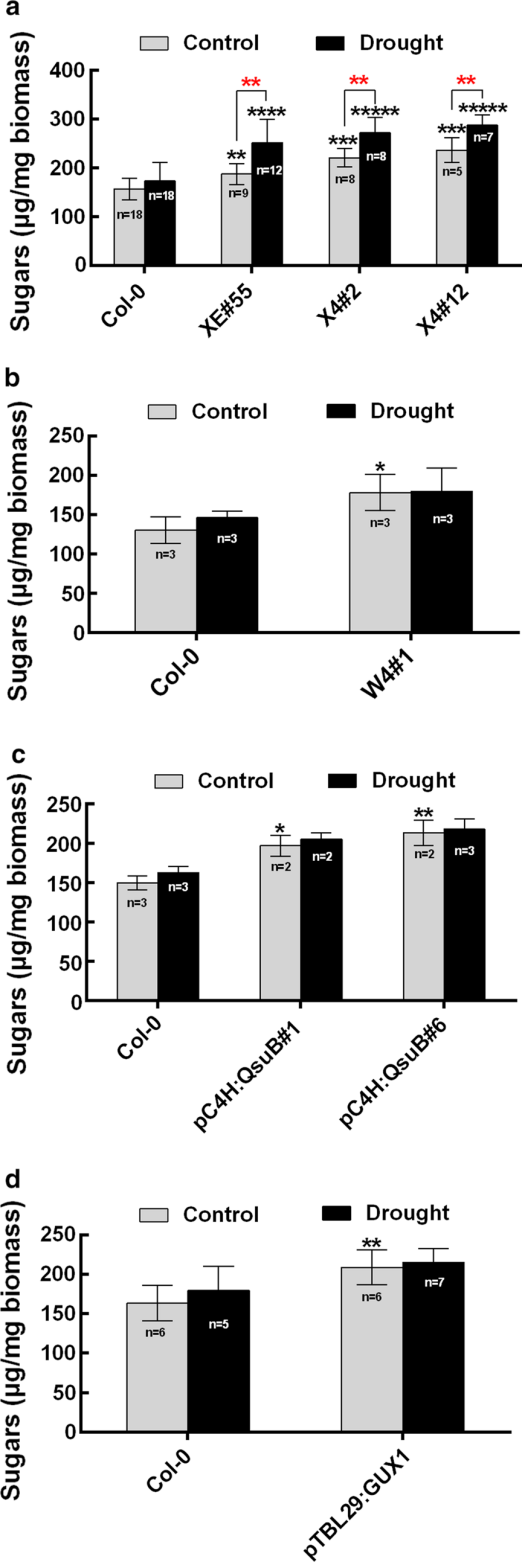
Saccharification analysis of the wild-type and engineered plants in response to drought treatment. **a** Saccharification analysis of XE#55, X4#2 and X4#12. **b** Saccharification analysis of W4#1. **c** Saccharification analysis of pC4H:QsuB#1 and pC4H:QsuB#6. **d** Saccharification analysis of pTBL29:GUX1. Hot water treatment of dry stem material was followed by 72 h of saccharification with the CTec2 (Novozymes) enzyme mixture. Values show average ± SD (*n* shown in the figure). Black asterisks indicate significant differences in the well-watered condition compared to the wild type, and red asterisks indicate significant differences between well-watered condition and drought stress condition (*t* test, *P < 0.05, **P < 0.01, ***P < 0.001)

Given that several of the engineered Arabidopsis lines had improved drought tolerance, we wanted to investigate whether the saccharification of biomass from engineered plants and wild type was affected under drought stress. For these experiments, the plants were drought treated after bolting (Additional file 2) to obtain stem biomass for analysis. Upon hot water pretreatment and after 72 h of enzymatic digestion with the Cellic CTec2 enzyme cocktail under normal conditions, the engineered lines XE#55, pC4H:QsuB, W4, and X4 showed an increase in saccharification yield compared to that of the wild type, which is similar to the previous reports [8, 12, 22] (Fig. 5a–c). We found that pTBL29:GUX1 also has an increased sugar release compared to that of the wild type (Fig. 5d). Xiong et al. [17] observed an increased sugar release in their study of the same plants, but it was not significant. However, they had fewer replicates and used a different saccharification protocol. It seemed that drought stress could cause a small yet not significant increase in sugar release in the wild type (Fig. 5). In contrast, we found that the engineered plants with low xylan, i.e. XE#55, X4#2, and X4#12 plants, release much more sugar after drought stress compared to the non-stressed condition (Fig. 5a).

### Engineered plants with low xylan have increased cell wall galactose under drought stress

**Fig. 6:**
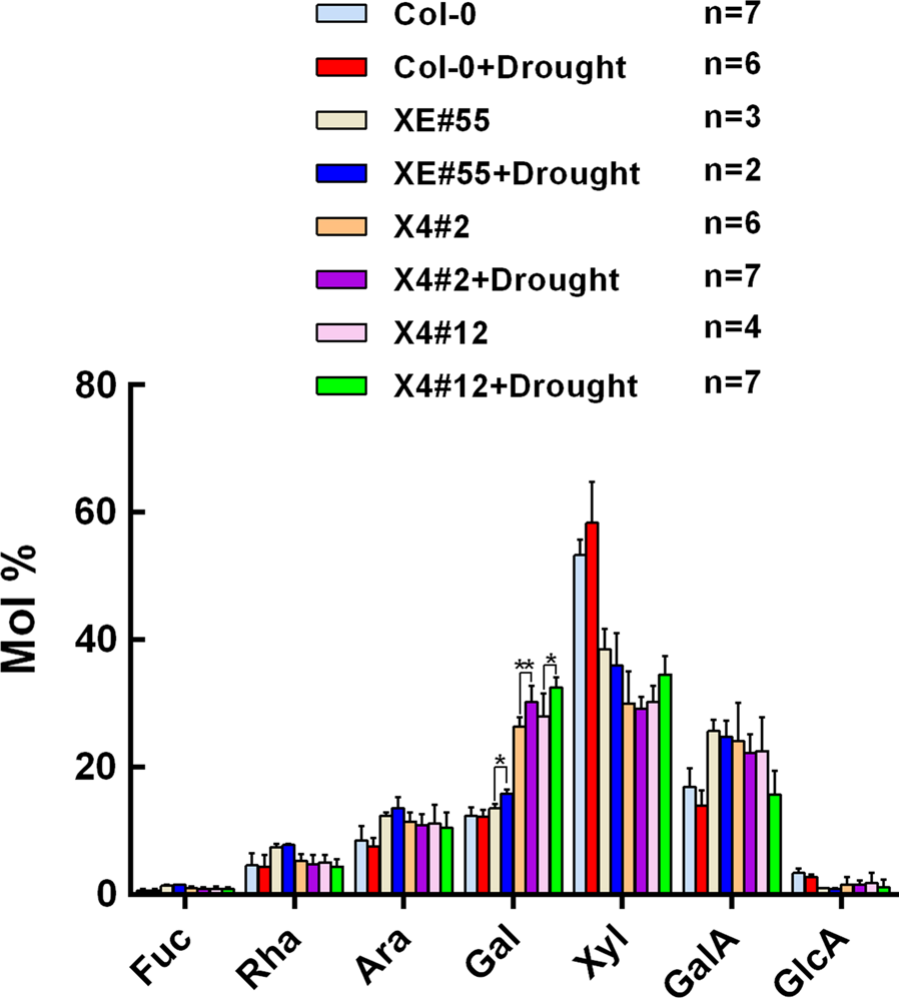
Monosaccharide composition of the cell wall from the Col-0 and engineered plants with low xylan under drought stress treatment. Cell wall material (AIR) was prepared from fresh main stem, hydrolyzed with 2 M trifluoroacetic acid (TFA), then analyzed by high-performance anion-exchange chromatography. Values show average ± SD (*n* shown in the picture). Asterisks indicate significant differences between well-watered and drought stress conditions (*t* test, *P < 0.05, **P < 0.01). *Fuc* fucose, *Rha* rhamnose, *Ara* arabinose, *Gal* galactose, *Xyl* xylose, *GalA* galacturonic acid, *GlcA* glucuronic acid

Because the engineered lines XE#55 and X4 exhibit an increased saccharification yield after drought stress (Fig. 5a), we wanted to analyze changes in their cell walls. Monosaccharide composition analysis of the main stems revealed that the galactose content of XE#55 and X4 under stress conditions show a significant increase of 15–17% compared to normal conditions, whereas the wild-type plants (Col-0) showed no difference (Fig. 6). In general, the changes in cell wall composition in response to drought were very minor.

## Discussion

To utilize the renewable and abundant biomass resources for the production of commodity chemicals and biofuels, several key hurdles need to be surpassed to reach economic viability. Processing of the biomass and conversion into biofuels is expensive and substantial improvements have been achieved by optimizing these steps. However, modifying the composition of the biomass itself can contribute to the economic viability of the overall process, and key improvements would include a reduction in the lignin content, increase in the ratio of hexoses to pentoses, and reduction of polymer-derived processing inhibitors [11]. Recently, several plant biotechnological approaches were used to engineer plants to address the recalcitrance and improve the composition [8, 12, 22, 32, 33]. Though no negative impacts were observed on plant growth and biomass in many engineered plants grown under optimal conditions, it is unclear whether there would be a positive or negative impact on plant growth and biomass under the environmental conditions in the field. Often there is a concern that adverse effects on plant growth and development are not obvious unless the plants are challenged by stress. Among such varied stresses, drought stress is the main abiotic stress that severely affects plant growth and yield [25]. Thus, it is important to study the physiological processes that underlie stress injury and the adaptation of cell wall-modified plants to drought stress.

Consistent with previous reports that the Arabidopsis xylan mutant *irx14* has a drought-tolerant phenotype, our results revealed that the plants engineered with low xylan are more tolerant compared to the wild type [34] (Fig. 1a and b). However, in contrast to the *irx14* mutants, which show a decreased growth [35], the xylan-engineered plants in the present study do not have a growth penalty. Our study also found that the engineered plants with low lignin (pC4H:QsuB, X4 and W4) and low xylan degree of acetylation (pTBL29:GUX1) showed drought tolerance (Fig. 1; Additional file 2). These engineered plants exhibit reduced levels of water loss, which is necessary for plants to survive under drought stress (Additional file 1). Second, some drought-responsive genes of these drought-tolerant plants are more highly induced in these engineered plants when they are exposed to drought stress (Fig. 4). It is important to note that all the plants in this study showed normal growth in well-watered conditions, and they did not exhibit an irregular xylem phenotype (Additional file 5). All the measurements of ABA content, drought-response gene expression, and stomatal opening confirmed that in the well-watered condition, the engineered plants were indistinguishable from the wild type.

ABA levels in plants significantly increase in response to drought stress, resulting in the closure of stomata and thereby reducing water loss through transpiration from leaves [26, 28, 29, 36]. Therefore, it is particularly interesting to investigate whether the low lignin, low xylan, and low degree of xylan acetylation contribute to the responses related to ABA signaling. In the present study, three lines of evidence clearly suggest that the engineered plants with low lignin improve drought tolerance in an ABA-dependent manner. First, the engineered plants showed obvious decreases in the cotyledon greening percentages compared with the wild type when exposed to ABA treatment (Fig. 2). Second, the plants engineered for low lignin accumulated higher ABA contents compared to wild type in response to drought (Fig. 3a). Finally, the stomatal closure in plants engineered for low lignin was more sensitive to exogenous ABA compared to that in the wild type (Fig. 3b and c). In contrast, the engineered plants with low xylan content and a low degree of xylan acetylation have similar ABA sensitivity and ABA levels compared to the wild type (Fig. 3a and Additional file 4). Taken together, these results indicate that the engineered plants with low lignin content confer the adaptation to drought tolerance in an ABA-dependent pathway. However, the engineered plants with low xylan content and low xylan acetylation have an improved survival rate under water deficit in an ABA-independent pathway. Why the plants respond in this way is presently unclear. The low lignin plants in this study all incorporate QsuB expression, which leads to metabolite change in the plants, e.g. the accumulation of protocatechuic acid [22]. It is possible that this somehow affects the drought response. There may also be changes in the way the engineered plants activate cell wall integrity responses, and we think it is possible that the altered cell walls lead to a stronger activation of cell wall sensors during drought stress [37–40].

Moreover, we found that the plants engineered for low xylan content have significantly increased saccharification efficiency under drought stress (Fig. 5a). Drought stress increases the galactose content of engineered plants with low xylan (Fig. 6), but this cannot directly explain the increased sugar release since the enzyme cocktail we used did not contain galactanases.

## Conclusions

Few prior reports have been written about the drought response of plants that were engineered to have improved cell wall composition. However, such studies are important for the biosystem design of bioenergy crops and will assist the predictable engineering of such crops. One study showed decreased lignin and improved saccharification by downregulation of phenylalanine ammonia lyase in *Brachypodium distachyon*, yet drought tolerance was not different from that of wild-type plants [41]. This result suggests that the drought tolerance we have found in the lignin-engineered plants is unique to our approach for lignin reduction. Ultimately, environmental resilience must be evaluated in the field and in the actual crop species, such as sorghum, switchgrass, and poplar. Nevertheless, this study inspires confidence that it is possible to engineer plants with improved cell wall composition and which are not merely able to grow well under non-stressed conditions; they are so resilient to stress that they can even perform better under certain stress conditions.

## Methods

### Plant material and growth conditions

All Arabidopsis wild-type and engineered plant lines used are in the ecotype Columbia (Col-0). The engineered plant lines used in this study (XE#55, pC4H:QsuB#1, pC4H:QsuB#6, W2#5, W4#1, W4#2, X4#2, X4#12 and pTBL29:GUX1) have been described before and references are listed in Table 1. All the genotypes were first grown together to maturity to ensure that the seeds used in the subsequent experiments had the same age and growth history. This was important to reduce variation in the results. Arabidopsis seeds were surface sterilized and sown on solid medium containing 0.5× Murashige and Skoog salts including vitamins (1/2 MS medium) (Sigma-Aldrich) and 2% (w/v) sucrose. Following stratification (48 h, 4 °C, in the dark), plates were transferred to a growth room (22 °C, 100–200 µmol/m^2^ s, 14 h light/10 h dark, 60% humidity).

### Assay for rate of water loss

The leaf water loss rate was assessed in detached whole rosettes of comparable size from 4-week-old plants. The rosettes were weighed immediately, and then put on a laboratory bench (50% relative humidity) and weighed again at different times. The rate was calculated on the basis of the initial mass of the rosette.

### Cotyledon greening assay

To determine the response of seeds to ABA inhibition of germination, seeds harvested at the same time were used for cotyledon greening. About 100 seeds of wild-type and engineered plants were sown on 1/2 MS medium containing 2% sucrose with or without 0.5 µM ABA. The seeds were stratified by putting the plates at 4 °C for 48 h in the dark, and then placed at 22 °C under light conditions. The number of germinated seedlings with green cotyledons was counted after 6 days at 22 °C.

### Root elongation assay

Five-day-old seedlings grown in 1/2 MS medium containing 2% sucrose were transferred to 1/2 MS medium, supplemented with or without different concentrations of mannitol for 10 days.

### Drought tolerance assays

For drought tolerance assay of plants in the rosette stage, seedlings from wild-type Col-0 and engineered plants were used. One-week-old seedlings were transferred to soil in 6-cm pots for 2 weeks under standard growth conditions. These plants were subjected to progressive drought by withholding water for about 2 weeks and then re-watered for 2 days after which the percentage of surviving, green plants was scored.

For drought tolerance test of plants in a later bolting stage, 1-week-old seedlings were transferred to soil and grown for about 4 weeks under standard growth conditions. When the inflorescence stems were about 0.5 cm tall, the plants were subjected to progressive drought by withholding water for 10 days and re-watered for 2 days after which the percentage of surviving, green plants was scored.

### ABA content

ABA content was measured as described by [42]. Briefly, 3-week-old plants were subjected to drought treatment as described above. After 9 days of water withholding, rosettes were collected from drought-stressed and well-watered plants. About 100 mg of leaves was used for ABA quantification by high-performance liquid chromatography–mass spectrometry [42].

### Measurement of stomatal closure in response to ABA

Stomatal closure assays were conducted as described previously [29]. Leaves of 4-week-old plants were floated in a solution containing 1 mM CaCl_2_, 20 mM KCl, and 5 mM MES, pH 6.15, and exposed to light (room temperature) for 2.5 h. Subsequently, 10 µM ABA or ethanol control was added to the buffer and incubated for another 2.5 h. Then the abaxial epidermal strips were quickly peeled to make slides and photographed in random sequence with a microscope [29]. The stomatal opening widths and lengths were measured using the ImageJ program and the width/length ratio was used as index of stomatal opening.

### Real-time PCR analysis

Wild-type and engineered plants were subjected to drought treatment when 3 weeks old as described above. After about 9 days of water withholding, rosette leaves were collected from drought-stressed and well-watered plants, then frozen in liquid nitrogen and stored at − 80 °C. Total RNA was extracted using the RNeasy Plant Mini Kit (Qiagen). RNA was first treated with Dnase I (Qiagen), and first-stand cDNA synthesis was performed using the iScript cDNA Synthesis Kit (Bio-Rad) according to the manufacturer’s protocol. RT-PCR was performed using SYBR Select Master Mix (Applied Biosystems) on diluted (five times) cDNA using a StepOne Plus Real-Time PCR System (Applied Biosystem 7500). The internal control and various drought-responsive genes’ primers are listed in Additional file 6. Expression levels for all candidate genes were determined using the 2^−ΔΔCT^ method [43].

### Cell wall preparation and monosaccharide composition analysis

When the inflorescence stems were about 4 cm tall, the plants were subjected to progressive drought by withholding water. After about 10 days of water withholding, the inflorescence stems were collected from both well-watered and drought-stressed plants. Alcohol-insoluble residue (AIR) was prepared as described by [8]. Dried AIR (2 mg) was hydrolyzed in 2 M trifluoroacetic acid (TFA) at 121 °C for 1 h and analyzed by high-performance anion-exchange chromatography (HPAEC) on an ICS-5000 instrument (Thermo Fisher Scientific) equipped with a CarboPac PA20 (3 mm × 150 mm, Thermo Fisher Scientific) analytical anion-exchange column, PA20 guard column (3 mm × 30 mm), borate trap, and a 500-pulsed amperometric detector (PAD), as described previously [44]. Glucose was not calculated due to the presence of residual starch.

### Hot water pretreatment and saccharification

When the length of the inflorescence stem reached about 4 cm, the plants were subjected to progressive drought by withholding water. After about 10 days of water withholding, the inflorescence stems were collected from both well-watered and drought-stressed plants, then dried at 50 °C for 3 days. The saccharification assay was described by [8]. In brief, dried stem material (5 mg fine powder) was mixed with 200 µl of water and then incubated with shaking for 30 min at 30 °C, followed by incubation for 1 h at 120 °C. The samples were allowed to cool. For enzymatic saccharification, a mixture of 5 mg/ml tetracycline and Cellic CTec2 enzyme mix (Novozymes, Denmark) in 0.1 M citrate buffer, pH 5.0 was added to the pretreated samples, followed by incubation at 50 °C for 72 h at 800 rpm. After saccharification, sugars in the supernatant were quantified with a reducing sugar assay using dinitrosalicylic acid reagent (DNS) as described [8].

### Microscopy

The basal 2.5 cm of main stems were fixed and sectioned as described [12]. The sections were stained with 2% phloroglucinol–HCl as described [7]. Pictures were taken using a DM6 B epifluorescence microscope (Leica) equipped with a C11440 Hamamatsu camera monitored by the LAS X software (Leica).

### Sequence IDs

The promoters and coding sequences used in the gene constructs relate to the following IDs: TBL29, At3g55990; GUX1, At3g18660; VND7, At1g71930; IRX7, At2g28110; C4H, At2g30490; QsuB, YP_001137362.1; Irx5, At5g44030; Irx8, At5g54690; CesA7, At5g17420; GalS1, At2g33570; UGE2, At4g23920; URGT1, At1g76670; Actin1, At2g37620; RD29A, At5g52310; RD29B, At5g52300; DREB2A, At5g05410.

## Additional files

**Additional file 1.** The rate of water loss from detached rosettes.

**Additional file 2.** Survival rate of plants in the flowering stage in response to drought stress.

**Additional file 3.** Growth of seedlings under osmotic stress.

**Additional file 4.** Germination of engineered plants with low xylan acetylation and low xylan content in response to 0.5 µM ABA.

**Additional file 5.** Stem cross-sections of wild-type and engineered plants.

**Additional file 6.** Genes and primers used in qRT-PCR.
