# Supplementary material for: Increased drought tolerance in plants engineered for low lignin and low xylan content
Source: Biotechnol Biofuels. 2018 Jul 18;11:195. doi: 10.1186/s13068-018-1196-7 (PMC6050699; doi:10.1186/s13068-018-1196-7)
Supplement: Supplementary file 1 — Additional file 1. The rate of water loss from detached rosettes. [file 13068_2018_1196_MOESM1_ESM.pdf]

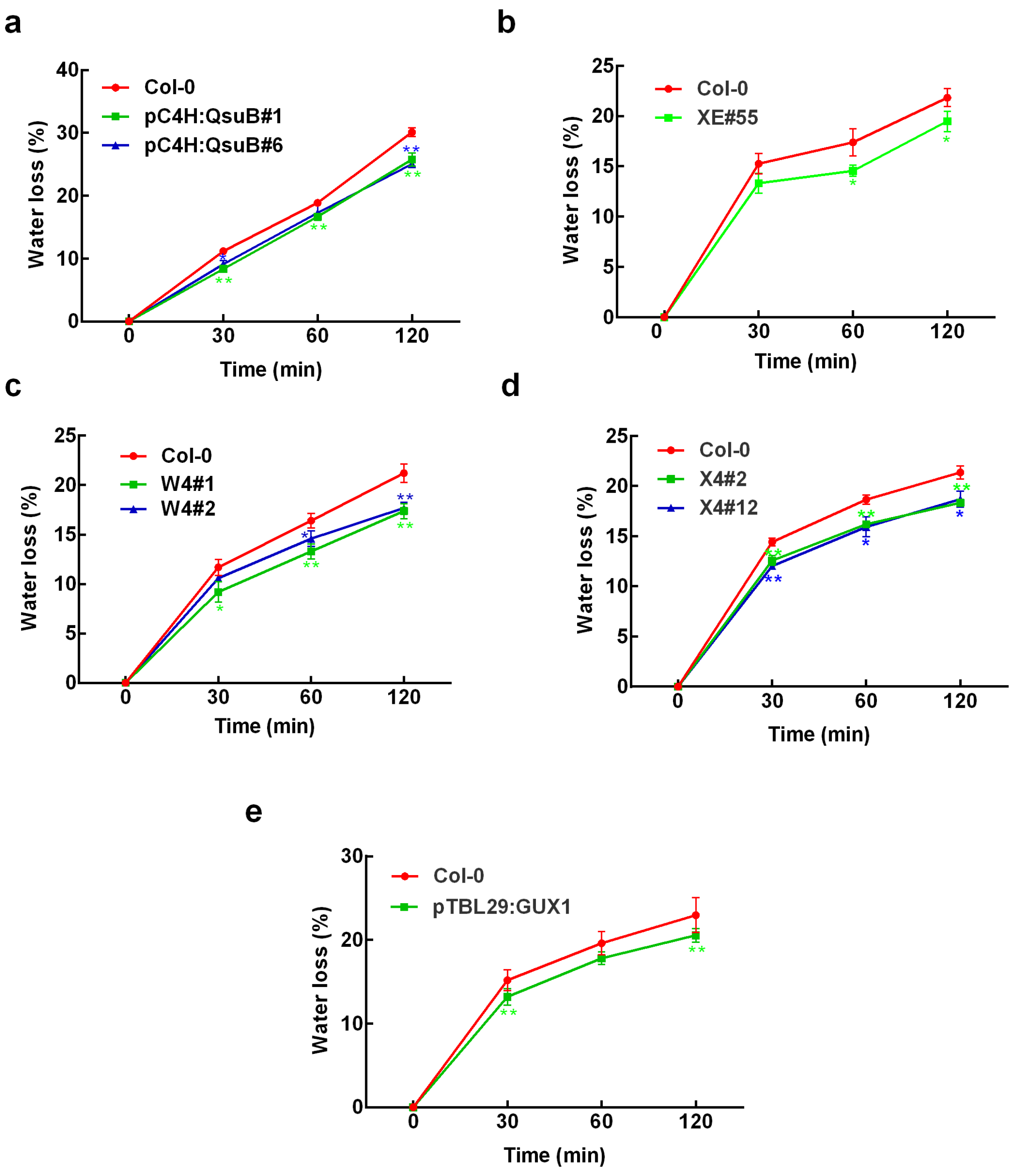

Additional File 1. The rate of water loss from detached rosettes. Rosettes were obtained from four-week-old plants and placed at a laboratory bench (approximately 50% relative humidity. Weight loss was calculated based on the initial mass. Values show average ± SD (n>3). Asterisks indicate significant differences from the wild type using Student's t-test (*P < 0.05; **P < 0.01).
