# Supplementary material for: Increased drought tolerance in plants engineered for low lignin and low xylan content
Source: Biotechnol Biofuels. 2018 Jul 18;11:195. doi: 10.1186/s13068-018-1196-7 (PMC6050699; doi:10.1186/s13068-018-1196-7)
Supplement: Supplementary file 2 — Additional file 2. Survival rate of plants in the flowering stage in response to drought stress. [file 13068_2018_1196_MOESM2_ESM.pdf]

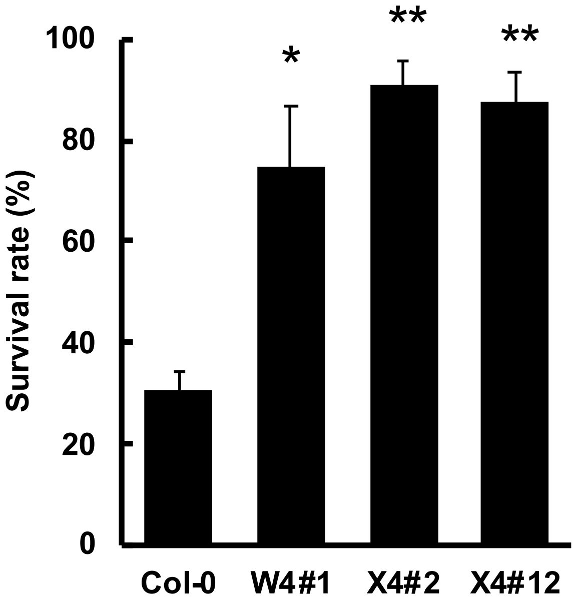

Additional File 2. Survival rate of plants in the flowering stage in response to drought stress. Water was withheld when inflorescence stems were 0.5 cm tall. After seven days the plants were rewatered and the number of surviving, green plants was scored. The experiment was repeated at twice with 18-24 plants of each genotype in each experiment. Values show average ± SD. Asterisks indicate significant differences from the wild type (t-test, *P < 0.05; **P < 0.01).
