# Supplementary material for: Increased drought tolerance in plants engineered for low lignin and low xylan content
Source: Biotechnol Biofuels. 2018 Jul 18;11:195. doi: 10.1186/s13068-018-1196-7 (PMC6050699; doi:10.1186/s13068-018-1196-7)
Supplement: Supplementary file 3 — Additional file 3. Growth of seedlings under osmotic stress. [file 13068_2018_1196_MOESM3_ESM.pdf]

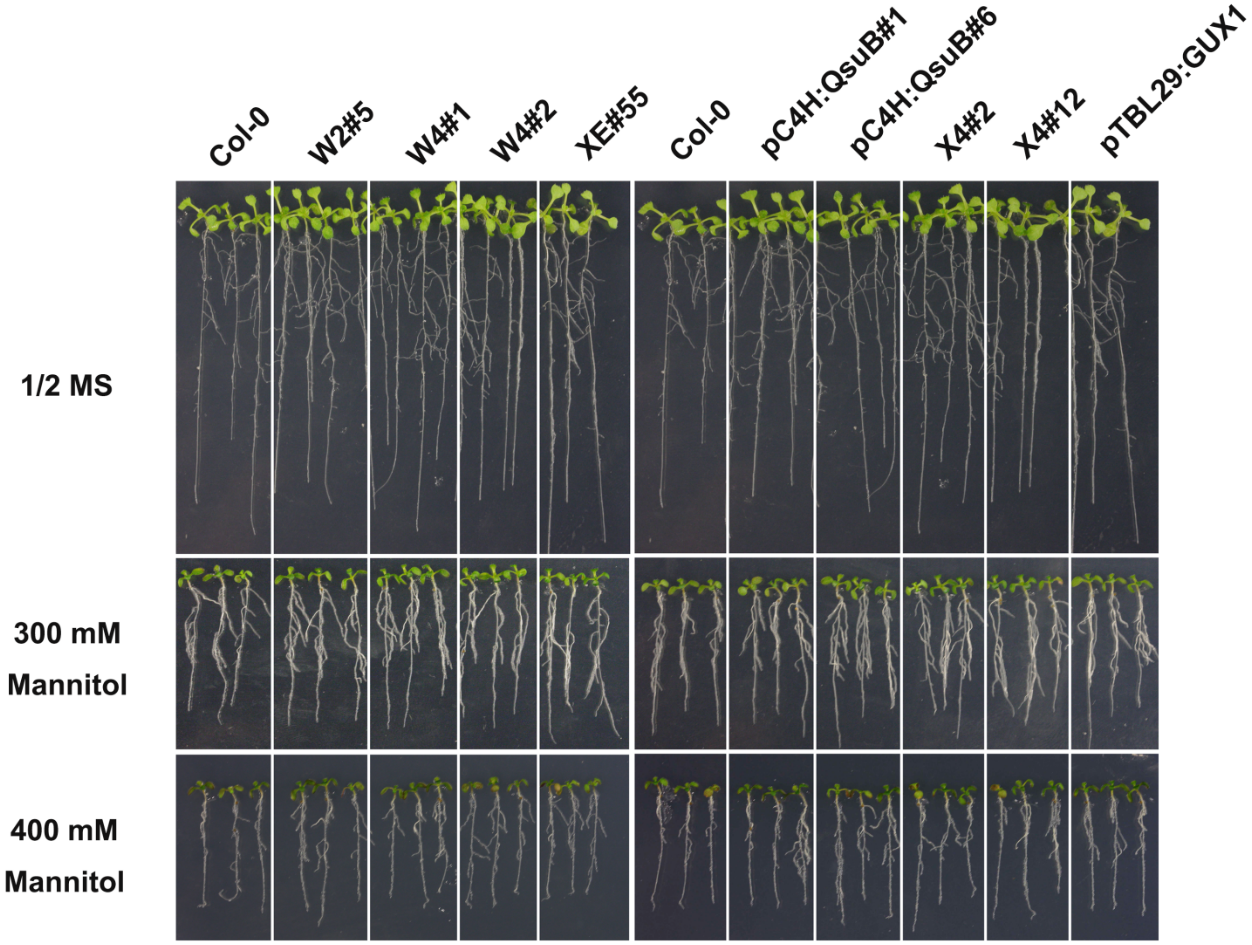

Additional File 3. Growth of seedlings under osmotic stress. Five-day-old seedlings grown in 1/2 MS medium containing 2% sucrose were transferred to 1/2 MS medium, supplemented with or without different concentrations of mannitol for ten days. The experiment was repeated at least three times. All the engineered plants showed root growth similar to wild-type plants under these conditions.
