# Supplementary material for: Increased drought tolerance in plants engineered for low lignin and low xylan content
Source: Biotechnol Biofuels. 2018 Jul 18;11:195. doi: 10.1186/s13068-018-1196-7 (PMC6050699; doi:10.1186/s13068-018-1196-7)
Supplement: Supplementary file 4 — Additional file 4. Germination of engineered plants with low xylan acetylation and low xylan content in response to 0.5 µM ABA. [file 13068_2018_1196_MOESM4_ESM.pdf]

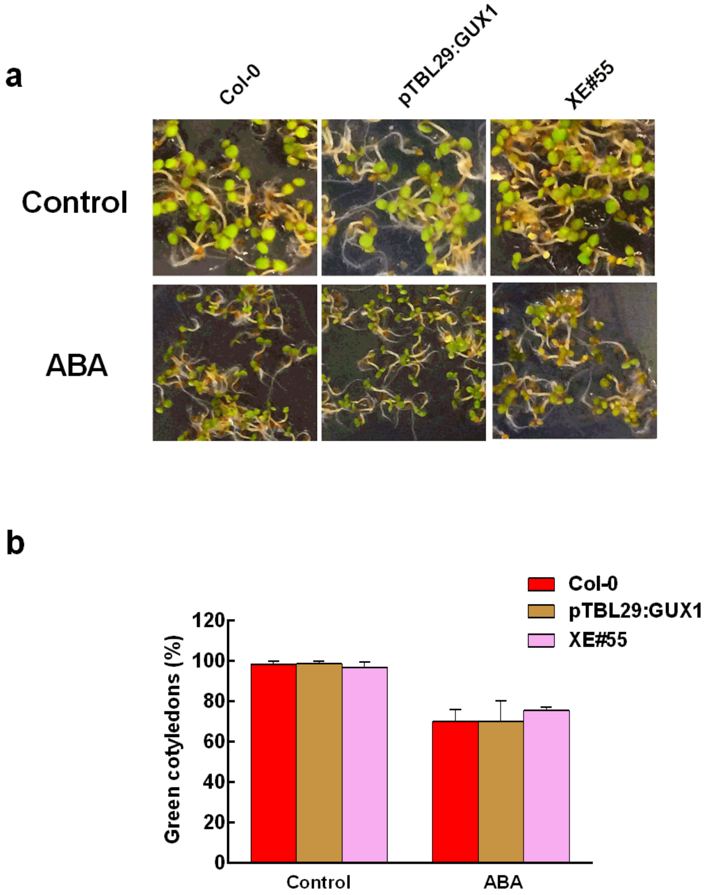

Additional File 4. Germination of engineered plants with low xylan acetylation and low xylan content in response to 0.5 μM ABA. (a) The wild-type and engineered plants were sowed on 1/2 MS medium containing 0 and 0.5 μM ABA. Photographs were taken after six days of growth. (b) The percentage of seedlings with green cotyledons was measured after six days. Values show average ± SD. Each experiment included at least 100 seeds, and three independent experiments were conducted. The engineered plants do not show any significant differences compared to wild type.
