# Supplementary material for: Increased drought tolerance in plants engineered for low lignin and low xylan content
Source: Biotechnol Biofuels. 2018 Jul 18;11:195. doi: 10.1186/s13068-018-1196-7 (PMC6050699; doi:10.1186/s13068-018-1196-7)
Supplement: Supplementary file 5 — Additional file 5. Stem cross-sections of wild-type and engineered plants. [file 13068_2018_1196_MOESM5_ESM.pdf]

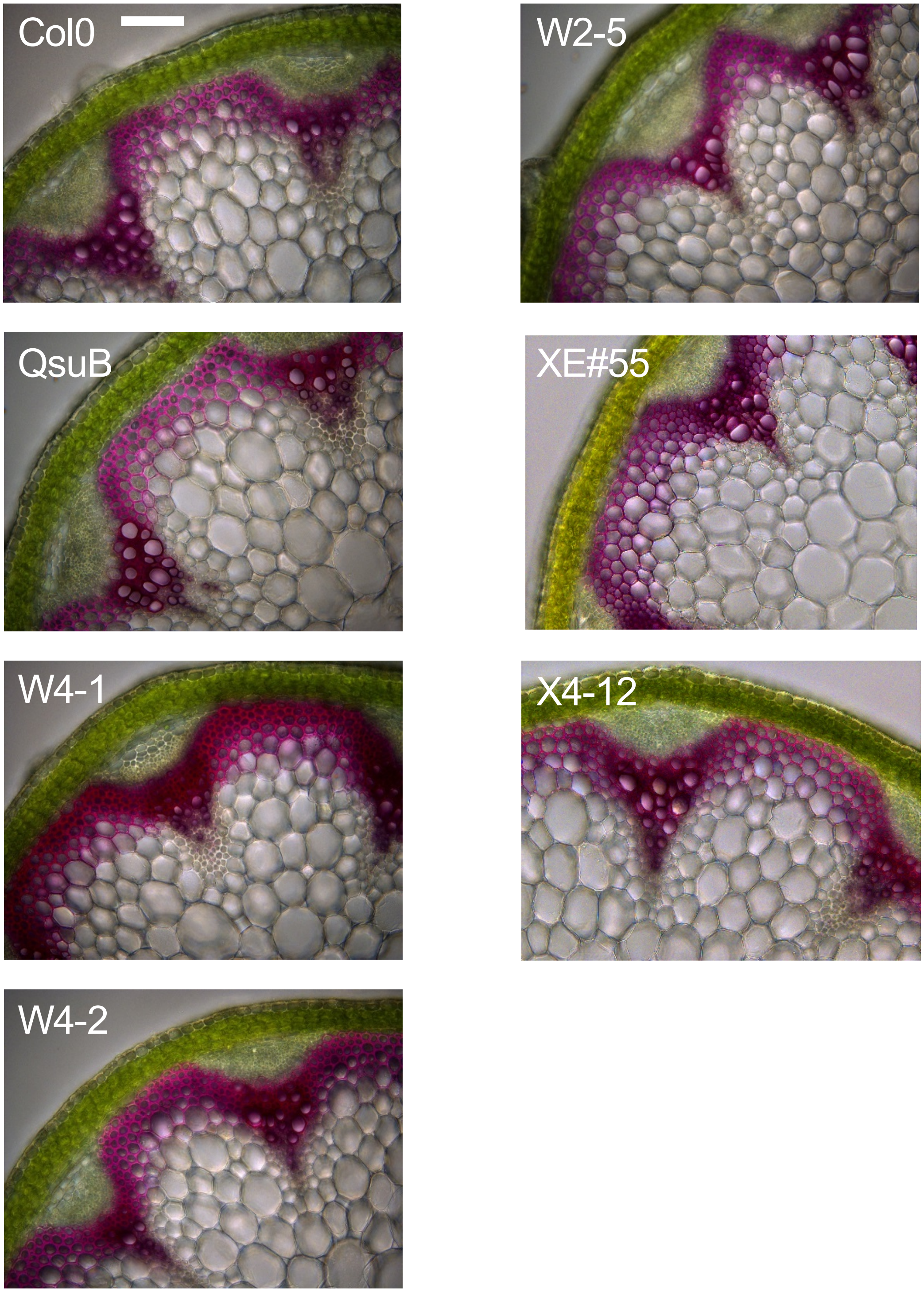

Additional File 5. Stem cross-sections of wild-type and engineered plants. The sections were stained with phlorogucinol-HCl, which stains lignin. Unlike many lignin and xylan mutants, none of the engineered plants exhibit irregular xylem. Scale bar 0.1 mm.
