# Supplementary material for: Increased drought tolerance in plants engineered for low lignin and low xylan content
Source: Biotechnol Biofuels. 2018 Jul 18;11:195. doi: 10.1186/s13068-018-1196-7 (PMC6050699; doi:10.1186/s13068-018-1196-7)
Supplement: Supplementary file 6 — Additional file 6. Genes and primers used in qRT-PCR. [file 13068_2018_1196_MOESM6_ESM.docx]

**Additional File 6.** Genes and primers used in qRT-PCR.

| **Genes** | **Primer** | **Sequence (5’-3’)** |
| --- | --- | --- |
| *Actin 1* | Forward | GTCCTCGACTCTGGAGATGGTGTG |
| (AT2G37620) | Reverse | GTCGTACTACCGGTATTGTGCTCG |
| *RD29A* | Forward | GCACCAGGCGTAACAGGTAA |
| ([AT5G52310](https://www.arabidopsis.org/servlets/TairObject?id=132264&type=locus)) | Reverse | TCGGAAGACACGACAGGAAA |
| *RD29B* | Forward | CTTGGCACCACCGTTGGGACTA |
| (AT5G52300) | Reverse | TCAGTTCCCAGAATCTTGAACT |
| *DREB2A*  (AT5G05410) | Forward | TCGTCCCCTATAGATTGTGTTGT |
|  | Reverse | GCCACAGTAGTACCGTCACC |
